# The potential utility of hybrid photo-crosslinked hydrogels with non-immunogenic component for cartilage repair

**DOI:** 10.1038/s41536-021-00166-8

**Published:** 2021-09-10

**Authors:** Yili Wang, Levinus Hendrik Koole, Chenyuan Gao, Dejun Yang, Lei Yang, Chunwu Zhang, Huaqiong Li

**Affiliations:** 1 Joint Centre of Translational Medicine, The First Affiliated Hospital of Wenzhou Medical University, Wenzhou, People’s Republic of China; 2 Zhejiang Engineering Research Center for Tissue Repair Materials, Joint Centre of Translational Medicine, Wenzhou Institute, University of Chinese Academy of Sciences, Wenzhou, Zhejiang People’s Republic of China; 3 School of Biomedical Engineering, School of Ophthalmology & Optometry and Eye Hospital, Wenzhou Medical University, Wenzhou, Zhejiang Province People’s Republic of China; 4 Orthopaedic Institute, The First Affiliated Hospital, Soochow University, Suzhou, People’s Republic of China

**Keywords:** Tissue engineering, Implants

## Abstract

Finding a suitable biomaterial for scaffolding in cartilage tissue engineering has proved to be far from trivial. Nonetheless, it is clear that biomimetic approaches based on gelatin (Gel) and hyaluronic acid (HA) have particular promise. Herein, a set of formulations consisting of photo-polymerizable Gel; photo-polymerizable HA, and allogenic decellularized cartilage matrix (DCM), is synthesized and characterized. The novelty of this study lies particularly in the choice of DCM, which was harvested from an abnormal porcine with α-1,3-galactose gene knockout. The hybrid hydrogels were prepared and studied extensively, by spectroscopic methods, for their capacity to imbibe water, for their behavior under compression, and to characterize microstructure. Subsequently, the effects of the hydrogels on contacting cells (in vitro) were studied, i.e., cytotoxicity, morphology, and differentiation through monitoring the specific markers *ACAN*, *Sox9*, *Coll2*, and *Col2α1*, hypertrophy through monitoring the specific markers alkaline phosphatase (*ALP*) and *Col 10A1*. In vivo performance of the hydrogels was assessed in a rat knee cartilage defect model. The new data expand our understanding of hydrogels built of Gel and HA, since they reveal that a significant augmenting role can be played by DCM. The data strongly suggest that further experimentation in larger cartilage-defect animal models is worthwhile and has potential utility for tissue engineering and regenerative medicine.

## Introduction

One of the major challenges of the biomaterials science & engineering field is to come forward with a robust biomimetic neo-cartilage construct that can be applied by orthopedic surgeons to repair articular cartilage defects^1,2^. Undoubtedly, one of the underlying reasons is that articular cartilage has a complex inhomogeneous structure. Going from the articular surface to the subchondral bone three zones can be discerned, each having a distinct structural build-up. The superficial zone consists of collagen fibers running parallel to the surface of the joint, has relatively high water content, accommodates chondrocyte cells which have a flat geometry and which are relatively abundant, and contains proteoglycan 4, a lubricating protein. The middle zone holds collagen fibers which are thicker and much more randomly organized as compared to those in the superficial zone. Less chondrocyte cells are encountered (per mm^3^), and the shape of these cells is spherical rather than flat. In the deep zone, which essentially marks the transition between cartilage and the underlying bone, collagen fibers are thickened further, and they run vertically. The chondrocyte cells are larger than cells in the middle zone, they are present in piled arrangements and surrounded by a so-called peri-cellular matrix consisting of collagen-IV. Also, glycosaminoglycans (GAGs) (such as hyaluronic acid [HA]), glycoproteins, and Ca^2+^ are found in the deep zone, which is also commonly referred to as “calcified cartilage”^3,4^.

The chondrocytes residing in cartilage—regardless of their location, shape, and size—express a highly specialized phenotype, able to synthesize extracellular matrix. However, chondrocyte-driven in situ matrix production is slow, and self-healing in the case of articular defects is usually disappointing if not absent^5^. This explains the need for biomimetic plugs to fill the defect surgically. Ideally, such a plug should fit in the defect, show no mismatches with the surrounding tissues (in terms of structure, presence of chondrocyte cells, matrix mechanical properties, water content, etc.), and engage rapidly in integration with surrounding tissues. Perhaps the closest match to such an ideal cartilage plug is provided by autologous cartilage (graft transplantation). Indeed, cases of successful functional repair and integration between autologous cartilage grafts and the surrounding tissue have been described^6,7^. Nonetheless, autologous grafting is linked to formidable practical problems, such as unavailable tissue, problems at the donor site (e.g., infection), and physical/mechanical mismatches of the plug, especially if it was harvested in a non-load-bearing area^8^. An alternative strategy is based on the use of allografts i.e., problems related to surgery and removal of tissue at a donor site are then avoided. Other advantages are that: (i), cartilage can be harvested from a similar load-bearing tissue and (ii), bigger grafts can be harvested, i.e., larger defects can be filled^9^. A tough problem typically associated with allografts is that immunogenic rejection may occur, and this is why allografts by no means provide an adequate solution to the problem. Integration between allografts and lateral (surrounding) cartilage on one hand and subchondral bone, on the other hand, is usually insufficient, and the fit and mechanical performance of the graft is often inferior.

Two major other approaches exist. One is based on cell-based therapy in which mesenchymal stem cells are introduced in the cartilage effect aiming at their differentiation in chondrocytes^10,11^. This approach falls outside the scope of this paper, and will not be discussed further. The second is based on the use of a biomaterial which can be synthetic or stem from a biological origin. The biomaterial construct must be designed in such a manner that the construct gets populated with chondrocyte cells postimplantation; in such cases, we speak of “tissue engineering”^12,13^. Besides, of course, the biomaterial construct should match the donor site in terms of structure (varying physical-mechanical properties and water content according to the three zones, vide supra). So far, this has also proved a huge challenge. A promising biomaterial is chitosan, which brings the advantages that it is non-immunogenic, having a structural resemblance to GAGs, and being a substrate for lysozyme^14,15^. Chitosan has also been implicated to support the production of collagen II in vivo^16^. Yet another strategy is based on the use of Gel and HA^17–19^. Note that Gel is essentially collagen that is partially hydrolyzed, and HA is a natural constituent of cartilage (vide supra)^20,21^. A particularly interesting approach, in our opinion, is based on the use of photocurable derivatives of Gel and HA. This means that the cartilage cavity is to be filled with a biomimetic liquid prepolymer mixture that fills the defect irrespective of its irregular shape. The prepolymer is subsequently crosslinked in situ through short-term irradiation with ultraviolet light (photo-polymerization). An example of this approach is the work of Levett et al., who studied mixtures of photocurable Gel and photocurable HA in the presence of chondroitin sulfate^22^. The latter is a bioinspired additive that was assumed to further support cell growth and integration of the material with its surroundings.

Inspired by the work of Levett et al., we designed and prepared a new set of bioderived and bioactive hydrogel formulations consisting of (i) photocurable Gel, (ii), photocurable HA, and (iii) allogenic DCM that was obtained from a porcine source^23^. The novelty of our approach lies in the use of DCM in our formulations. We hypothesized that growth factors and GAGs, which are still present in the DCM, will help to (a) regenerate the native extracellular matrix and (b) populate the implant with functional chondrocytes^24–26^. Of note, the allogenic DCM material that was used in this work was obtained from gene-knockout pigs that were deficient in an α-1,3-galactose gene. It is well known that the presence of α-1,3-galactosyl glycoprotein molecules on the surface heterogeneous endothelial cells gives rise to immune reactions and hence to (hyperacute) rejection of allografts^27^. Hence, the use of allogenic DCM lacking α-1,3-galactose was hypothesized to (i) circumvent severe implant-associated inflammation which is known to be associated with the use of allogenic cartilage matrix and (ii) bring essential growth factors and GAGs to the cartilage regeneration site, thus fostering a suitable microenvironment for chondrocyte cells. We conducted a comprehensive study of DCM-loaded photo-crosslinked Gel-HA hydrogels in six different formulations. We studied these new hydrogels through spectroscopy (NMR, FTIR), and we determined the most pertinent physicochemical properties, such as water absorption, behavior under compression, and porosity/microstructure. Furthermore, we assessed (i) absence/presence of cells in the DCM; (ii) degree of cytotoxicity of the diverse formulations (Quantitative-iT^Tm^ PicoGreen^®^ dsDNA assay) and Live-Dead staining and cytoskeleton staining; (iii) morphology of cells present in the hydrogels, using fluorescence microscopy and staining with acridine orange (AO), ethidium bromide (EB) (Live-Dead assay), rhodamine, and DAPI; (iv) differentiation of cells present in the hydrogels through monitoring the specific markers *ACAN*, *Sox9*, *Coll2*, *Col2α1*, *ALP*, and *Col 10A1* thereby utilizing real-time polymerase chain reaction using SYBR Green Master Mix; (v) in vivo performance of the hydrogel (rat knee cartilage defect model), thereby using the scaffold to fill the cartilage defect. The new data expand our understanding of hydrogels built of Gel and HA, and their potential utility in cartilage healing.

## Results

### Analysis of DCM

**Fig. 1:**
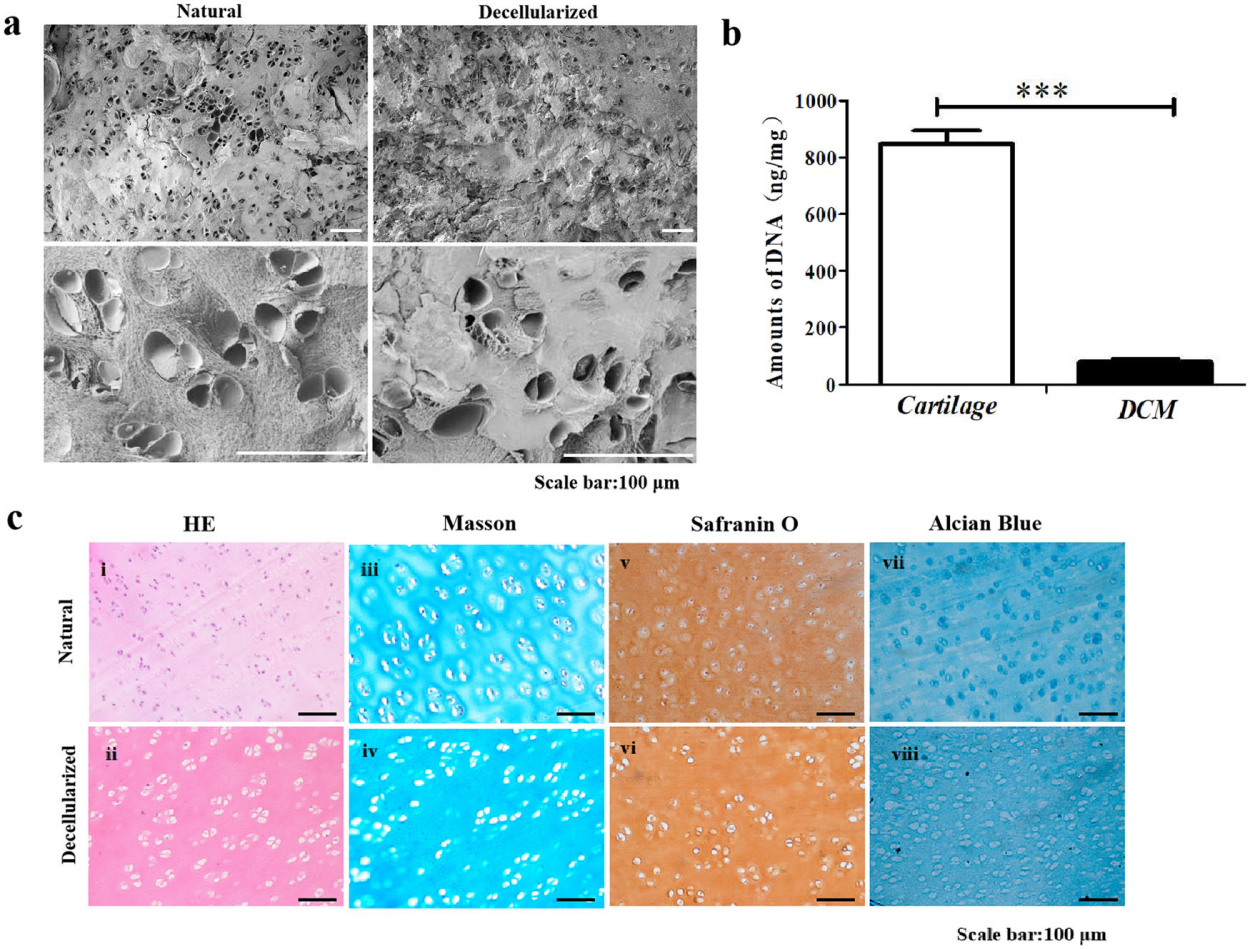
Characterization of natural and decellularized cartilage tissue. **a** SEM observation of cartilage. **b** Data from the DNA content analysis. **c** Photo micrographs of microtome slices of cartilage pre and post decellularization. Error bars: ± SD, ***p < 0.001. Scale bar = 100 μm.

SEM revealed that pristine cartilage and DCM have very similar morphologies; typical lacunae (with diameter in the range 20–30 mm) were very clearly visible at higher magnification (Fig. 1a). DNA analysis showed that the DNA content of the natural cartilage is as high as 813.9 ± 26.3 ng/mg vs. 85.8 ± 5.7 ng/mg for its decellularized counterpart; this is a statistically significant difference (*p* < 0.001). This further substantiates our conclusion that the decellularization procedure was effective. We note that the DNA content found for the pristine cartilage is higher than the value of 270 ng/mg as was found by Madry et al. for the adjacent cartilage^28^. DCM material was screened as thoroughly as possible for the absence/presence of cells. Although α-1,3-Gal knockout pigs were the source of the cartilage, cells or remnants thereof can still ignite immune reactions. Two different methods were used to assess the absence or presence of cells and analysis of ECM components: (i) histology with classical tissue staining (H&E, Masson, Safranin O, and Alcian blue), and (ii) DNA content. Results are compiled in Fig. 1b. H&E staining showed a marked difference for treated and untreated cartilage in the sense that almost no nuclei could be detected for the treated material and the extracellular matrix is preserved (red), compare (i) and (ii) in Fig. 1c. The Masson staining results revealed that collagen in the extracellular matrix of the cartilage tissue after decellularization was retained; micrographs were virtually identical for the sample ± treatment, compare (iii) and (iv) in Fig. 1c. Histology could not prove that cytokines and other important components are still present and functional/intact in the ECM. This is hoped for, as this would likely facilitate differentiation of invading cells into chondrocytes, and thus provide favorable conditions for cartilage repair/regeneration. The native cartilage and DCM were stained with Safranin O and Alcian blue to detect the cartilage matrix and proteoglycans. Safranin O staining (v) and Alcian blue staining (vii): many chondrocytes with blue-stained and red-stained peripheral matrices can be observed, respectively. The Safranin O (vi) and Alcian blue staining (viii) results revealed that the presence of cartilage matrix and proteoglycans was consistent with the native cartilage in Fig. 1c.

### Swelling of hydrogels 1–8

**Fig. 2:**
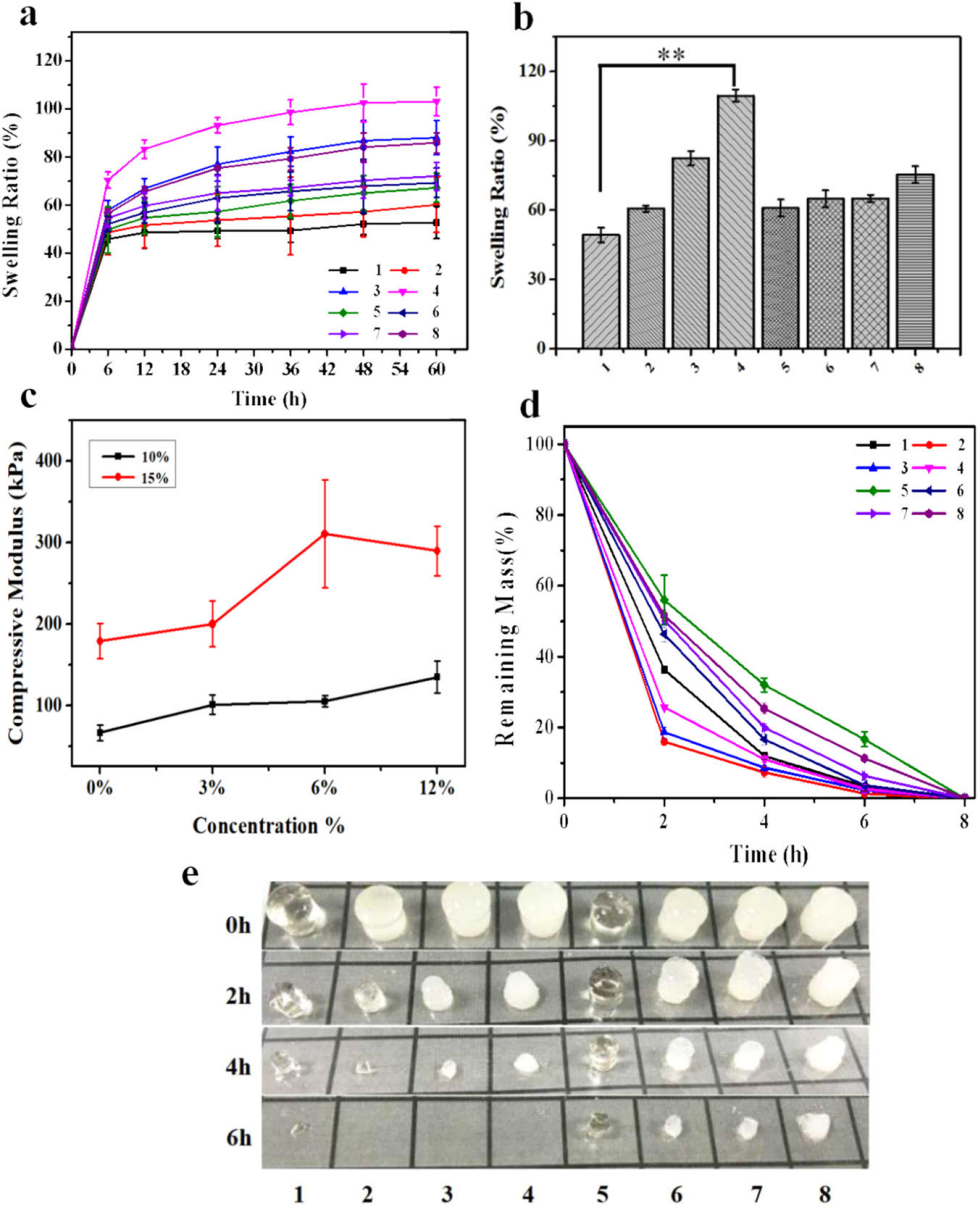
Analysis of physical properties of hydrogels 1–8. **a** Relative mass increase (%) as function of time due to water uptake and swelling of materials 1–8 during incubation in PBS buffer at 37 °C. In both series of materials (A and B), the formulations with the largest content of DCM (i.e., 4 and 8, respectively) show the fastest and highest degree of swelling. **b** Relative mass increase of materials 1–8 at equilibrium. The water content increase in the Series A and B, showing that added DCM contributes to the hydrophilic character of the hydrogels. **c** Apparent Young’s modulus of the hydrogels 1–8. In both Series A and B, it is seen that increased DCM content correlates with increased stiffness. **d**, **e** Experimental data on enzymatic degradation of the materials 1–8 (by type-IIcollagenase). The data reveal the biodegradable nature of the materials. (Error bars: ± SD, *p < 0.05, **p < 0.01, ***p < 0.001).

Our data on aqueous swelling of the materials **1**–**8** in PBS (37 °C) are displayed in Fig. 2a as plots of the relative mass increase vs. time. Water uptake varies between -roughly- 40 and 100%, and complete equilibration of the most hydrophilic versions requires 60 h at least. The most water-imbibing i.e., most hydrophilic version is material **4** (10%GelMA/1%HAMA/12%DCM, absorption 110% ± 3% by mass), whereas materials **1** and **5** (10%GelMA/1%HAMA and 15%GelMA/1%HAMA) absorb the lowest amounts of water (45–60% by mass). Clearly, the DCM additive contributes substantially to the degree of water absorption: in **Series A**, water absorption increases from 48% (**1**) to 110% (**4**), see Fig. 2b. This effect of added DCM is less obvious in **Series B** in which water absorption increases only marginally, from 60% (**5**) to 75% (**8**), see Fig. 2b.

### Hydrogel behavior under compression

Data from our experiments in which cylindrical samples of materials **1**–**8** were compressed, are compiled in Table 1 and plotted in Fig. 2c. Note, however, that these experiments were done with so-called unconfined samples. Hence, water was squeezed out of the sample as compression proceeded; the process was essentially irreversible. The observed proportionality of stress and strain following the start of the experiment, therefore, defines a so-called “apparent E-modulus” which is to be distinguished from the E-modulus according to Hooke’s law.

**Table 1:** Experimental data from compression tests of the fully hydrated specimens of materials 1–8.

Material	Content of GelMA (%)	Content of DCM (%)	Apparent E-modulus (kPa; compression)
Series A			
1	10	0	66 ± 10
2		3	101 ± 12
3		6	105 ± 7
4		12	135 ± 20
Series B			
5	15	0	179 ± 22
6		3	200 ± 28
7		6	282 ± 66
8		12	305 ± 30

These data reveal that GelMA and DCM are both dominant factors governing the resistance of **1** through **8** to compression. Comparing **1** with **5**, **2** with **6**, **3** with **7,** and **4** with **8** reveals that stiffness goes up by a factor 2–3 when the GelMA content is increased from 10 to 15%. Analogously, stiffness increases by a factor of 2.8 in **Series A** and 1.7 in **Series B**, i.e., with increasing content of DCM from 0 to 12%.

### Degradation of hydrogels 1–8

Figure 2d, e summarizes experimental data on enzymatic degradation (type II collagenase) of materials **1** through **8**. Degradation is very fast under these experimental conditions, especially for materials **Series A**, which contain 10% GelMA; these materials totally dissolved within 6 h. Degradation of materials **Series B** is somewhat slower but still very fast; dissolution of these specimens was complete within 8 h. It can be concluded that higher content of GelMA is associated with longer time intervals required to complete degradation i.e., with enhanced stability. The content (%) of DCM in the material only has a minor impact on the kinetics of degradation.

### Microstructure studied by SEM

**Fig. 3:**
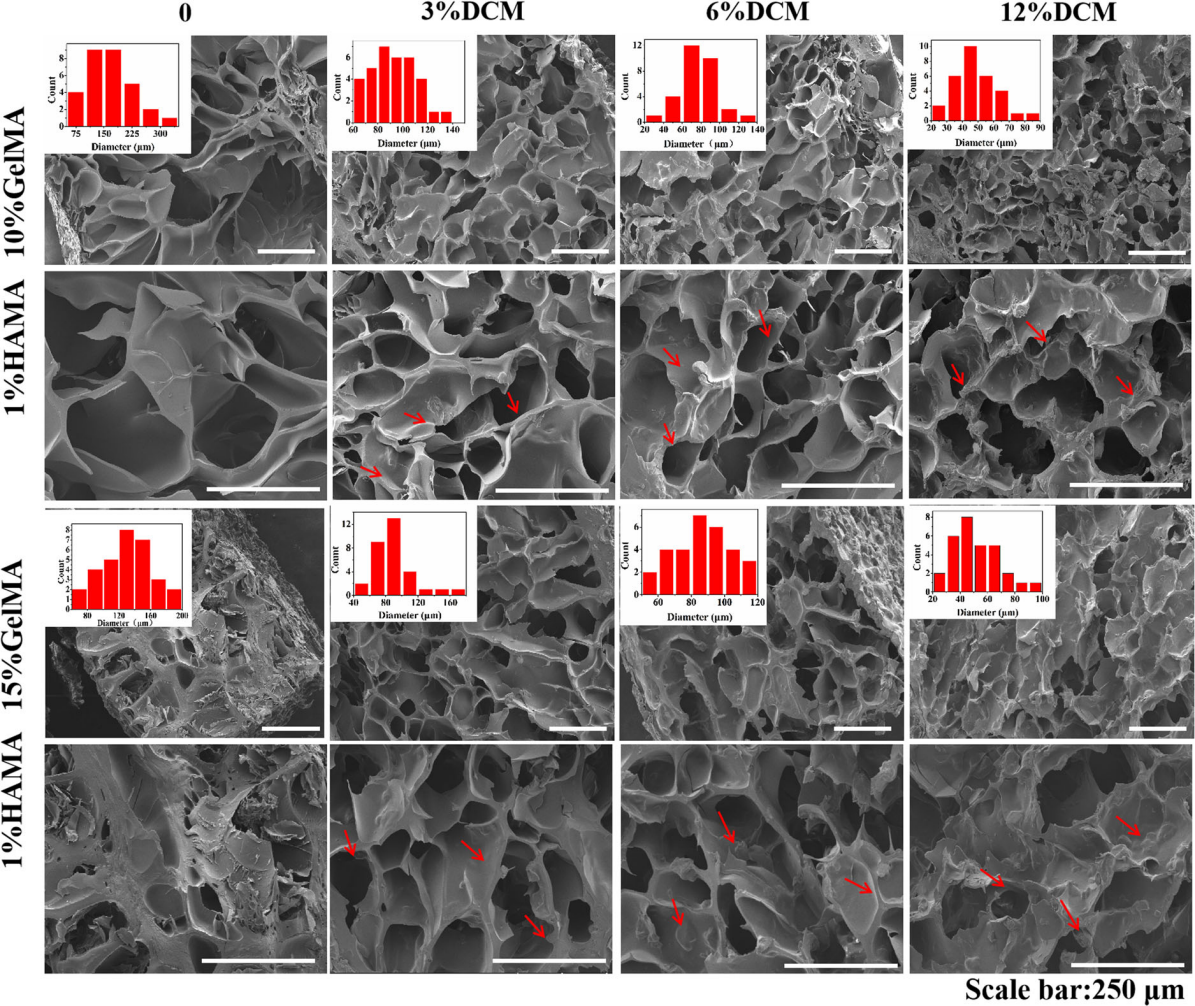
SEM micrographs, taken from fracture surface of the dry hydrogels 1–8. See text. DCM particles are designated by the red arrows. Scale bar = 250 μm.

Figure 3 shows representative SEM micrographs of **1**–**8**. All materials are markedly porous, which reflects the fact that each of the formulations that were subjected to photo-polymerization had high water content (78–90% by mass, see Table 2). We attempted to measure pore sizes and pore size distributions; the results are displayed in the inserts (red histograms). The data pointed at a bimodal distribution i.e., most of the pores have diameters in the ranges 140 ± 20 and 175 ± 25 μm. Materials **4** and **8**, having the highest content of DCM and the lowest content of water in their series (Table 2), appear to have the smallest pores (see the right column in Fig. 3). This was expected a priori.

**Table 2:** Compositions of the hybrid hydrogel.

	GelMA (mg)	HAMA (mg)	DCM (mg)	Water (mL; % by mass)	Abbreviation
Series A					
1	100	10	0	1.00; 90	10%GelMA/1%HAMA
2	100	10	30	1.00; 88	10%GelMA/1%HAMA/3%DCM
3	100	10	60	1.00; 85	10%GelMA/1%HAMA/6%DCM
4	100	10	120	1.00; 81	10%GelMA/1%HAMA/12%DCM
Series B					
5	150	10	0	1.00; 87	15%GelMA/1%HAMA
6	150	10	30	1.00; 84	15%GelMA/1%HAMA/3%DCM
7	150	10	60	1.00; 82	15%GelMA/1%HAMA/6%DCM
8	150	10	120	1.00; 78	15%GelMA/1%HAMA/12%DCM

### DPSCs in contact with hydrogels 1–8

**Fig. 4:**
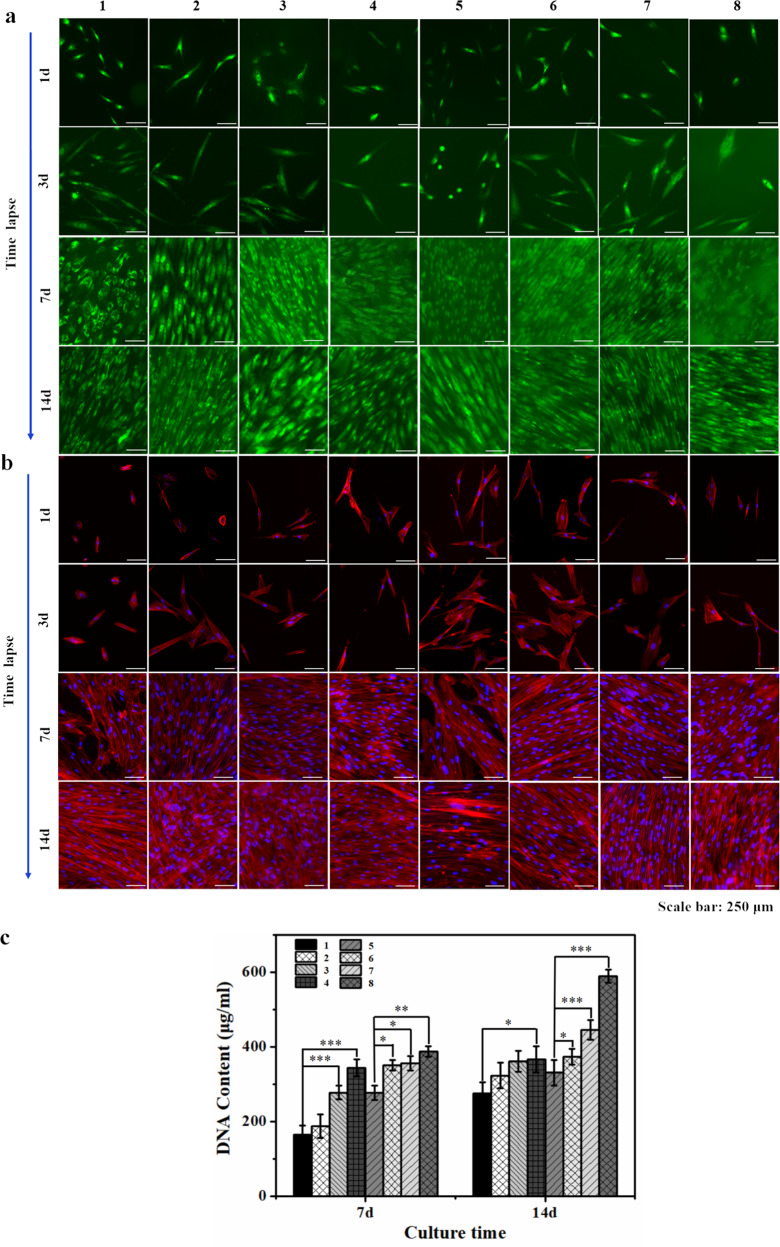
Analysis of the biocompatibility of hydrogels 1–8 in vitro. **a** Photo micrographs, taken by fluorescence microscopy, of adherent viable cells in contact with the materials of this study; Live–Dead assay. Horizontal: different materials (1–8); vertical: time (1, 3, 7, and 14 days post incubation). See text. **b** Photo micrographs, taken by fluorescence microscopy, of adherent viable cells in contact with the materials of this study; Stains: DAPI for cell nuclei and rhodamine for cytoskeleton. Axes like in panel **a**. **c** DNA content of the cells that adhered to materials 1–8 at two time points (7 days, left; 14 days, right). At both time points, cell viabilities are found to increase in the Series A and B, i.e., with increasing DCM content, see text. (Error bars: ± SD, *p < 0.05, **p < 0.01, ***p < 0.001). Scale bar = 250 μm.

Materials **1**–**8** present as thin surface coatings on well-bottoms of a 24-well plate (vide supra), were incubated with DPSCs as described above. Following the Live-Dead protocol (1, 3, 7, and 14 days after the time of incubation), we observed by fluorescence microscopy that the adherent cells were viable in all cases i.e., for all materials at all time points (ubiquitous green color; see Fig. 4a). Cell proliferation was still limited after 1 and 3 days of incubation; the adhered cells were separate and mostly adopting a spindle-like shape. After 7 days, however, cells had proliferated to confluency, as is seen in the middle and lower rows of Fig. 4a. This remained unchanged until 14 days of incubation. It should be noted that the observation of only green cells at all time points and for each of the materials **1**–**8** does not exclude the possibility that part of the cells died during the experiment. Such dead cells (which would stain red in Live-Dead) might have detached from the well bottoms and hence have remained unnoticed. More detailed insight into the morphology of the adherent alive cells was obtained through cell staining with rhodamine and DAPI. Figure 4b shows representative fluorescence micrographs of materials **1**–**8** at the three-time points. Details of cytoskeletons (stained red) and cell nuclei (stained blue) can be discerned, especially at time point 3 days post incubation when the cells were still separate. After 7 and 14 days, cells forced each other into close proximity as their numbers had increased markedly. Close inspection of Fig. 4b shows that proliferation has advanced farthest for the cells which were in contact with the materials that are richest in DCM, i.e., materials **3**, **4**, **7**, and **8**; it is of note that this is an observatory qualitative analysis only.

Quantitative data on the proliferation of the DPSCs in contact with materials **1**–**8** were derived from dsDNA content measurements (Quantitative-iT^Tm^ PicoGreen^®^ dsDNA assay). The results are summarized and plotted in Fig. 4c. **Series A** and **B** both show an increasing DNA content after 7 days (left side of Fig. 4c), revealing that the DPSCs divided faster when in touch with materials that were richer in DCM. The same trend can be observed after 14 days of incubation (right side of Fig. 4c). Evidently, GelMA also contributes to the aptitude of cells to proliferate. This becomes evident from comparing, for instance, DNA contents for **1** and **5**; **2** and **6**; **3** and **7** at day 7, and especially **4** and **8** at day 14.

### Quantitative analysis of chondrogenic differentiation

**Fig. 5:**
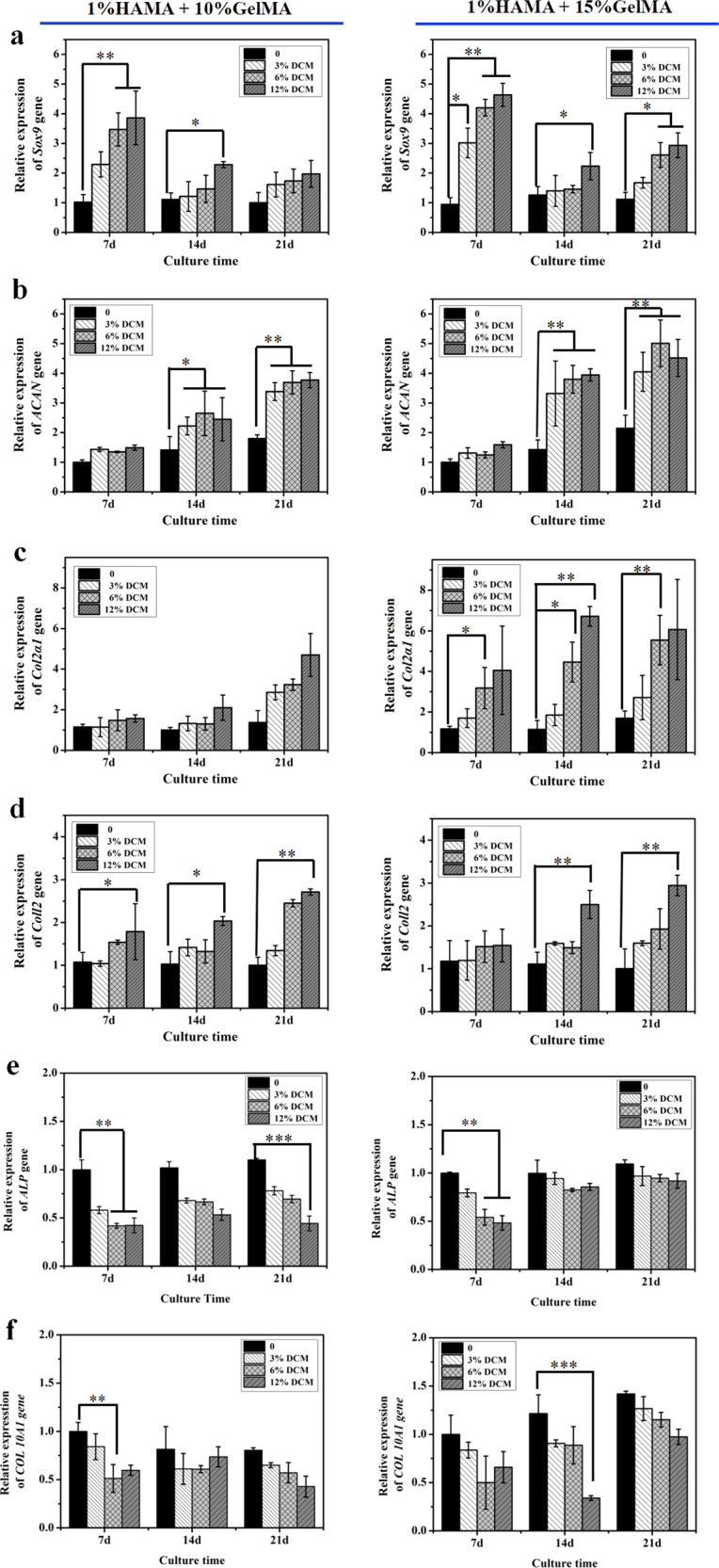
Quantitative analysis of chondrogenic differentiation and hypertrophy was evaluated by RT-PCR in vitro. The main markers of chondrogenic and hypertrophic differentiation include Sox9 (**a**), ACAN (**b**), Col2α1 (**c**), Coll2 (**d**), ALP (**e**) and Col 10A1 (**f**). Representative genes of cell chondrogenesis at different stages and marker genes of hypertrophy were selected. (Error bars: ± SD, *p < 0.05, **p < 0.01, ***p < 0.001).

The DPSCs seeded onto the hydrogels **1**–**8** were analysed for possible differentiation to chondrocytes and hypertrophy, at the time points 7, 14, and 21 days post incubation. Relative expression levels of the genes *ACAN*, *Sox9*, *Col2α1*, *Coll2*, *ALP*, and *Col 10A1* were determined by RT-PCR; the data were compiled in Fig. 5. *ACAN*, *Sox9*, *Col2α1*, and *Coll2* are specific indicators of chondroblast differentiation at different stages. *ALP* and *Col 10A1* are signature genes of hypertrophy^29,30^. We specifically looked for any upregulation of these genes at different time points. *Sox9*^28^, an early indicator of chondrogenic differentiation was found to be significantly upregulated at 7 days after cell seeding. The most obvious *Sox9* upregulation was found for materials **4** and **8** (containing the highest content (12%) of DCM); the increase in expression was up to 4.5-fold. The *Col2α1* and *Coll2* genes are known to be associated with the middle and late stages of chondrogenic differentiation. Indeed, upregulation of these genes was particularly clear at days 14 and 21 (a, b, c, and d in Fig. 5). For materials in which the GelMA content is 10% (**Series A**), the change of Col2α1 gene expression was not significant. However, for materials **5**–**8**, having a GelMA content of 15%, the gene upregulation amounts to sevenfold, which is significant. Upregulation of *Col2α1* increases the content of DCM is increased. The expression level of *Coll2* changes similarly to *Col2α1*. The *ACAN* gene is known to be a late indicator of chondrogenic differentiation. Our experimental data appear to be in line with this, showing upregulated *ACAN* on the time points 14 and 21 days, especially for the 15%-GelMA-containing materials (**Series B**). Of note, DCM content is not markedly influencing *ACAN*, expression of the gene is not statistically different for materials **2**, **3**, and **4** nor for materials **6**, **7**, and **8** at both time points i.e., 14 days and 21 days. The hypertrophy-associated genes *ALP* and *Col 10A1* are not obvious upregulated in the chondrogenic differentiation. In Fig. 5e, f, the expression of *ALP* and *Col 10A1* were slightly increased in 21 days cultured compared to 7 and 14 days of culture, see Fig. 5e, f. The Safranin O staining and Alcian blue staining also further confirmed that the material combined with DCM had a good ability to induce stem cell chondrogenic differentiation in vitro (Supplementary Fig. 3). Basophilic cartilage appears red in combination with the Safranin O and GAG combined with Alcian blue colored blue can be qualitative characterizations of chondrogenic differentiation for cells culturing on the biomaterials. Although DCM itself had signals on Safranin O and Alcian blue stainings, both colors gradually increased with increasing DCM content and culturing time. After 14 days, the colors of material **8** was the strongest, whereas, after 1 day, material **1** was the weakest (Supplementary Fig. 3).

### Performance of Series B in a rat knee cartilage defect model

**Fig. 6:**
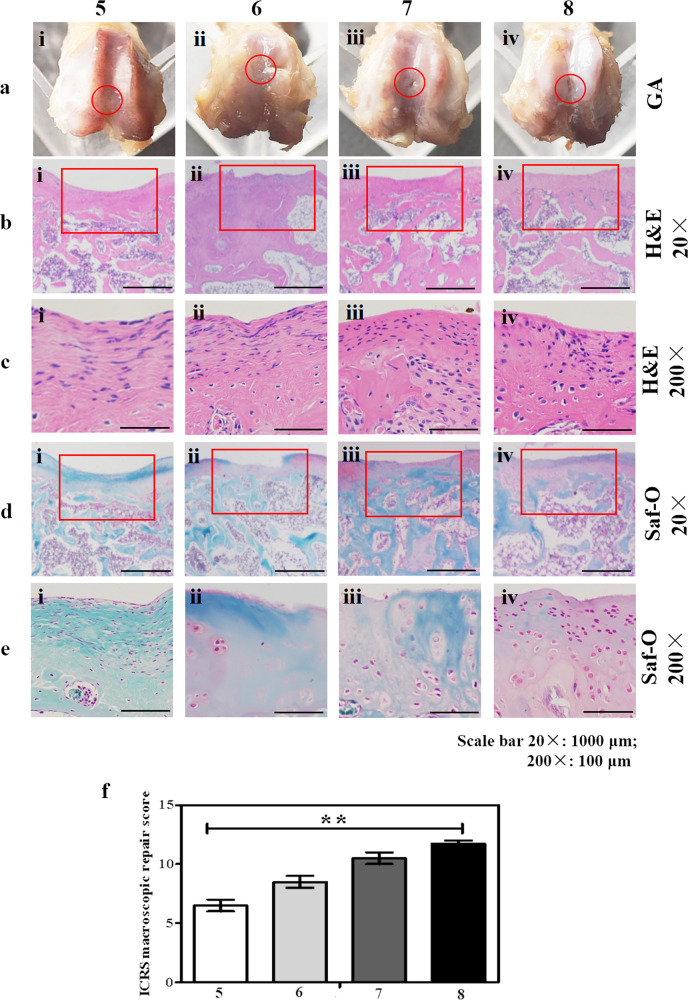
Data from in vivo experiments. **a (i**–**iv)** photographs of the cartilage layer on the femoral part of the knee joint (site of implantation), taken directly after sacrifice and opening of the knee joint. **b (i**–**iv)**, **c (i**–**iv)** representative tissue slices, stained with H&E, See text. **d (i**–**iv)**, **e (i**–**iv)** representative tissue slices, stained with Safranin O, See text. Red frames indicates the cartilage defect. **f (i**–**iv)** the ICRS macroscopic repair scores were quantified. (Error bars: ±SD, *p < 0.05, **p < 0.01, ***p < 0.001). Scale bar = 1000 μm (20×), Scale bar = 100 μm (200×).

Based on the in vitro data, we decided to evaluate the 15% GelMA-containing materials (**Series B**) in more detail in an in vivo cartilage repair model in the rat. The procedure as described by previous reference^31^ was followed with small modifications. Implantation of the specimens proceeded without difficulties in all 20 animals. Soon after their recovery, the animals appeared healthy, and behavior and mobility were normal. There were no casualties. At sacrifice (9 weeks post implantation), the incisions had healed in all cases; no signs of inflammation were noted. Gross inspection of the 20 sites of operation revealed that the defects in which the materials **6**, **7**, and **8** (i.e. the hydrogels consisting of 15% GelMA, 1% HAMA, and either 3, 6, or 12% DCM) were implanted appeared completely filled with tissue. The defect cavities in which the control material (**5**, only GelMA and HAMA, no DCM) was implanted, as well as the sham-defects, appeared only partially occupied. The second through fifth rows in Fig. 6 show representative tissue slices of the defect sites; the slices are vertical cross-sections through the implants and surrounding tissues. The second and third row show H&E-stained tissue slices; slices in the fourth and fifth rows were stained with Safranin-O. It must be noted here, that no distinction can be made between the materials that were implanted (so: GelMA, HAMA, and DCM), and the de novo extracellular matrix. In the second row (20×, H&E) it is seen that the cartilage surface is concave in (i), but much more space-filling in (ii)–(iv), which is in line with our gross inspection (Fig. 6b). In all cases, invasion of cells into the defect area is noted (third row, 200×, H&E). Cells are more abundant close to the surface, as compared with deeper regions; this is especially clear in (ii) and (iv) (Fig. 6c). Furthermore, the nuclei of the cells which reside close to the surface frequently adopt a more stretched and thin geometry, whereas cell nuclei from deeper positions appear more circular. This is reminiscent of the different abundance and shapes adopted by chondrocytes upon going from superficial cartilage to deep-lying cartilage (vide supra). The fourth row (20×, Safranin-O) again shows the implant regions for the different implant materials. The defect area appears to be only partly closed in (i) and (ii), but completely filled in (iii) and (iv) (Fig. 6d) i.e., for the materials **7** and **8** which have the highest content of DCM (6 and 12%, respectively). Note that the new tissues are markedly blue-colored in case of 0, 3, and 6% ECM; that is in (i)–(iii) (Fig. 6d) and (at higher magnification, 200×) in (i)–(iii) (Fig. 6e). The blue stain indicates the presence of bone rather than cartilage. Only in d(iv) and e(iv), so in the presence of 12% ECM, we see that the defect area became occupied by cartilage exclusively. The conclusion was drawn from the investigation and evaluation of the degree of defect repair, the degree of marginal integration, and the macroscopic appearance. The ICRS macroscopic scores were apparently higher in the material **8** group than the other three groups (Fig. 6f).

## Discussion

Repair of major cartilage defects, which may be caused by osteoarthritis or trauma, usually requires osteochondral transplantation i.e., surgical implantation of an autograft or allograft construct. The implant serves to fill up the damaged area, take over the functions of the original tissue, and stimulate healing. While such procedures frequently do result in relief of pain and improved quality of life for patients, the results on longer-term are mostly disappointing. This can, most likely, be attributed to poor postoperative integration of the grafts with surrounding cartilage and with the underlying bone. In addition, the physical-mechanical properties of autografts/allografts are almost inevitably in a mismatch with the properties of the original cartilage tissue. Even though cartilage has a high water content (~80% by mass), it has a complex anisotropic and inhomogeneous build-up that is extremely hard to mimic. The nonavailability of adequate graft materials for cartilage repair poses a longstanding, important, and tough problem in biomaterials science & engineering. This is probably best illustrated by the fact that a Pubmed search using the term “cartilage repair biomaterials”—conducted in November 2020—yielded no less than 702 hits during the last 5 years.

From the wealth of research data on cartilage repair, it can be distilled that synthetic polymers and hydrogels, whether biodegradable or not, cannot provide any adequate solution. We hypothesized, in part on the basis of data in the literature, that bioderived hydrogels built from (Gel and HA form a promising basis to research other formulations. The novelty of this study lies in our choice to add allogenic (porcine) DCM to the formulation. Note that the three components (Gel, HA, and DCM) are found in healthy cartilage. It was anticipated that DCM can bring growth factors and GAGs to the repair scene i.e., these components were expected to survive the treatments that are given to cartilage to remove the cells. The study took two further points into account: (i) use of photo-polymerizable derivatives of Gel and HA. This led to formulations that can be cured in situ, i.e., cavities with different geometries can then be filled and cured consecutively; (ii) use of decellularized porcine cartilage abstracted from a donor animal that is deficient in α-1,3-galactose gene, as a strategy to prevent rejection due to foreign-body immunogenicity.

Eight different hydrogel formulations consisting of GelMA, HAMA, and α-1,3-galactose- free DCM were subjected to a comprehensive analysis. The eight materials mimicked cartilage, not only in the sense that the three components occur in healthy cartilage, but also in the sense that the water content of the hydrogels is on par with that of healthy cartilage. First, experiments to establish the identity and purity of the eight materials were conducted. NMR was used to characterize GelMA and HAMA (Supplementary Fig. 1), FTIR spectra was used to analyze the composition of the materials (Supplementary Fig. 2). Histological staining and DNA content were used to assess the DCM. The main challenge of decellularization is to remove cellular components to reduce the immune response while maximally retaining the bioactive molecules. As we all know that cartilage tissue is very dense and it is difficult to remove cellular components. To overcome this difficulty, Weimin Fan et al^23^. used trypsin, deoxyribonuclease, and ribonuclease for decellularization, resulting in a large loss of GAG and collagen. In this study, the decellularization technology has been improved. The results of tissue staining and DNA content testing indicated that the cellular components were completely removed and the extracellular matrix (Stained in red), the collagen (stained in blue), the cartilage matrix (stained red), and the GAG (stained blue) are well retained (Fig. 1). After decellularization, the immunogenicity problem was resolved, and no inflammatory cells were found after implantation (Fig. 6).

Secondly, relevant physical properties such as uptake of water, behavior under compression, and degradation were measured. The swelling ability of hydrogels is an indication of the degree of hydrophilicity and the unique feature has been shown to influence cellular behavior. On the other hand, substrate stiffness has been shown to be important for the modulation of cellular behavior, such as the regulation of phenotypes. Khademhosseini et al.^32^ analyzed the properties of GelMA/HAMA and reported that the swelling rate of GelMA/HAMA ranges from 10 to 55%, and the compressive modulus was 73 kPa approximately. When we change the composition of the material, the addition of DCM makes the swelling rate up to 110% (Fig. 2b), the compression modulus range from 66 ± 10 kPa to 300 ± 30 kPa (Fig. 2c), which is a significant improvement. Buling Wu et al.^24^ acquired the extracellular matrix derived from allogenic decellularized bone marrow mesenchymal stem cell sheets and applied to the reconstruction of osteochondral defects. The results showed that pure ECM does not have adjustable mechanical properties and needs to be fixed by blood coagulation in vivo. The reason for these problems may be that pure ECM is too soft and difficult to shape. An attractive way to overcome this problem is to form composite scaffolds with synthetic polymers. Fen Li et al.^33^ developed an electrospun cartilage-derived extracellular matrix (cECM) and polycaprolactone (PCL) composite nanofiber membrane. The challenge of the cECM/PCL is degradation. In vivo experiment results showed that the material has not completely degraded in 24 weeks, which is not conducive to the growth of new tissues. In vitro, the hybrid hydrogel confirmed that the material can be completely degraded within 6 h (Fig. 2d); in vivo, the histological staining results indicated that the material has been completely degraded after 2 months, and the new cartilage is well connected to the original tissue (Fig. 6).

Thirdly, the behavior of dental pulp stem cells in contact with the materials was investigated (in vitro); no indications of any cytotoxic effects were obtained. DCM contains growth factors, GAGs, and proteins, provides the required microenvironment for cultured cells, is comparable to native tissues, and can also promote cell viability, proliferation, and differentiation. Eslaminejad et al.^34^ produced and evaluated the decellularized extracellular matrix hydrogel for cartilage regeneration derived from knee cartilage. The results showed that the internal structure of the hydrogel digested with acetic acid and pepsin was disordered. The DNA content data showed that the cell proliferation on natural cartilage and acellular cartilage is better than hydrogel. The internal structure of hybrid hydrogel studied in this paper was more orderly, the cavities were evenly distributed and the size is uniform (Fig. 3), which is conducive to the entry of cells and the transportation of nutrients. In addition, the experimental results of interaction with cells confirmed that a large number of living cells live on the material and the cells stretch on the surface of the materials (Fig. 4).

Fourthly and finally, the hydrogels of **Series B** were considered most promising on the basis of in vitro experiments. **Series B** was used in an in vivo (rat knee) cartilage defect model. In vivo, the results confirmed that the hydrogel materials had a good ability to induce cartilage repair, and the increase of DCM content further promoted the formation of new cartilage (Fig. 6), which was consistent with the results of RT-PCR (Fig. 5), Safranin O staining and Alcian blue staining in vitro (Supplementary Fig. 3).

We realize that the experimental data can by no means predict the suitability of these hydrogels for use in cartilage repair of human patients; the experimental models clearly have little predictive value. Yet, several nontrivial and surprising clues emerged from this study, and we believe these to be important: (i) dental pulp cells in contact with the materials showed differentiation into cartilage-type cells; (ii) defects in vivo were found to be filled with cartilage-like de novo tissue. This was non-vascularized tissue but nevertheless accommodated invaded cells and appeared integrated with surrounding cartilage. This study gave us an expanded understanding of biomimetic hydrogels on the basis of Gel and HA, and the apparent augmenting role of DCM from α-1,3-galactose deficient porcine origin. The data merit the continuation of research into these materials, especially through the use of larger in vivo models for cartilage repair (e.g., a sheep model). It is especially intriguing, in our opinion, that cartilage defects in such larger models can be filled with consecutive hydrogel layers, each having a (slightly) different formulation. Each layer can be cured by photo-polymerization. The layers should have increasing water content and decreasing stiffness upon going from deep-in-the-defect to superficial in order for the stack to be biomimetic. We anticipate that such experiments, which could not have been designed without the new data from this study, do come closer to clinical practice and will have more predictive value. Work along these lines is currently in progress in this laboratory.

In this study, in order to better simulate the composition of natural cartilage, GelMA/HAMA hydrogel materials with adjustable mechanical properties and the acellular cartilage matrix with excellent biocompatibility were selected and successfully constructed by UV cross-linking. GelMA and HAMA have some characteristics similar to cartilage tissue, such as high water content and elasticity, and have been widely used in regenerative tissue engineering and clinical medicine. As a relatively novel material, DCM has undergone decellularization treatment to eliminate the risk of immune rejection and retain most of the extracellular matrix and collagen fibers. The addition of DCM further enhanced the mechanical properties and has the advantage of inducing DPSCs to differentiate into cartilage. In vivo, cartilage defect experiments further confirmed that the hydrogel scaffold material with DCM can promote cartilage regeneration. Collectively, our results indicated that the combination of the three materials that mimic the composition of natural cartilage tissue could be a promising candidate for functional cartilage regeneration.

## Methods

### Precursor synthesis

Gelatin methacrylate (GelMA) was prepared according to Khademhosseini et al.^35^ with small modifications. Gel (20.00 g) was added to Dulbecco’s phosphate-buffered saline (DPBS, 200 mL) at 50 °C, and the mixture was stirred magnetically until complete dissolution. Methacrylic anhydride (MA, 16.00 mL) was added and stirring was continued for 3 h. More PBS (600 mL) was added and the solution was dialyzed against distilled water (40 °C, 2 h, membrane cut-off 12–14 kDa). Then, the solution was frozen (−80 °C, 24 h) and freeze-dried for 3 days (Boyikang Experimental Instrument Co. Ltd., Beijing, China). The yield of GelMA (white powder): 16.27 g. Analogously, HA (2.00 g) was added to phosphate-buffered saline (PBS, 200 mL) at 50 °C, and the mixture was stirred magnetically until complete dissolution. MA anhydride (2.00 mL) was added and stirring was continued for 24 h at 4 °C while maintaining the pH between 8 and 10 with the addition of sodium hydroxide (5 M). In addition to more PBS, dialysis, and freeze-drying were done as described above. The yield of HAMA (white powder): 1.84 g.

Cartilage (~200 g) was harvested from the ribs of an α-1, 3-Gal gene-knockout pig. The ribs were provided by Yifan Dai’s lab from Nanjing Medical University (Nanjing, China). Soft tissues and blood were removed carefully, the cartilage was washed repeatedly with PBS (500 mL, 3×) and cut into small pieces (~1 g each). The cartilage was further treated with 10 mM Tris-HCl (500 mL) at 45 °C for 24 h. The supernatant was removed and replaced by 0.25% (m/v) Trypsin (500 mL). The mixture was left to stand for 24 h at 37 °C. The supernatant was removed again and replaced by a protease inhibitor (500 mL, room temperature). The supernatant was refreshed once (after 1 h). The cartilage pieces were washed with PBS (500 mL, 3×), frozen (−20 °C) for 4 h, and ground with the automatic freezing grinding machine (Retsch, Haan, Germany), using a frequency of 20 sec^−1^, grinding cycle 3× (15 min each). Decellularized cartridge particles sized <40 μm were sieved out and stored at −80 °C. The animal experiment included in this part was in line with this ethical requirement, as approved by the Ethics Committee of Wenzhou Institute of the University of Chinese Academy of Sciences (WIUCAS 20033115 and 20200331).

### Preparation of the hybrid hydrogels

Materials (eight formulations) were prepared according to Table 2. Note that the GelMA content is either 10 or 15% (m/v); content of HAMA is 1% (m/v) invariably, and content of DCM is 0, 3, 6, or 12% (m/v). Note, furthermore, that materials **1**–**4** (containing 10% GelMA) are designated **Series A**, and materials 5–8 (containing 15% GelMA) are designated **Series B**.

To 1.00 g of each formulation, photo-initiator [5 mg, 0.5% (m/v); 2-hydroxy-4′-(2-hydroxyethoxy)-2-methyl acetone (Igracur 2959)] was added in the dark. A Teflon mold containing six holes (8.0 mm diameter, 1.0 mm depth) was used. First, the cavities were filled with mixtures **1** and **5**, and irradiated with UV light (365 nm, 18 mW/cm^2^, 2 min), thus yielding three samples per composition. This was repeated for compositions (**2** + **3**), (**4** + **6**), and (**7** + **8**) thus yielding 8 × 3 = 24 samples.

### Spectroscopic analysis by NMR and FTIR

Nuclear magnetic resonance (NMR) spectra (^1^H, 500 MHz) of GelMA and HAMA (i.e., the precursors of the cross-linking reaction) were run on a Bruker Avance Neo Spectrometer Durham, USA). D_2_O was used as the solvent, chemical shifts were referenced against residual solvent signals. Special notice was given to the resonances appearing in the range 5.2–5.7 ppm, as these stem from the vinylic protons of the tethered methacrylate groups. Fourier-transform infrared (FTIR) spectra in the frequency range 500–4000 cm^−1^ were recorded with a Fourier infrared spectrometer instrument (Bruker, Tensor II, Bremen, Germany). Samples were at room temperature. Data were analyzed with OriginPro 8.5. The results of NMR and FTIR were listed in Supplementary materials.

### Verification of cartilage decellularization

The matrix architecture of pristine and decellularized cartilage was studied with scanning electron microscopy (SEM; Hitachi SU 8010, Tokyo, Japan). The specimens were frozen (−80 °C), freeze-dried during 48 h (Boyikang Experimental Instrument Co. Ltd., Beijing, China), cracked, and sputter-coated with Pt, using a Leica EM ACE600 instrument (Leica GmbH, Wetzlar, Germany).

The Quant-iT^TM^ PicoGreen^®^ dsDNA assay kit (Invitrogen, Carlsbad CA, USA) was used to quantify residual DNA in the DCM material. Analyses of DCM (10 mg) and untreated cartilage (10 mg) were done in triplo. The materials were added to 0.1% Triton-X 100 (1 mL) and left to stand for 10 min. The supernatant was removed and replaced by TE buffer (200 mM Tris-HCl, 20 mM EDTA, pH 7.5), present in the kit. Then, PicoGreen detection reagent was added, following the instructions of the supplier. The absorbance was read at 520 nm using a microplate reader (ThermoFisher, Varioskan LUX, USA) for all six samples (three for DCM and three for pristine cartilage). Whether or not the genetic material was eliminated completely, and the cartilage matrix was retained was assessed further by hematoxylin & eosin (H&E) staining, Masson staining, Safranin O staining, and Alcian blue staining. DCM and cartilage specimens were placed in tissue fixative for 24 h, dehydrated (ethanol series 70% → 80% → 90% → 95% → 100%) and permeated in xylene. The completely infiltrated tissues were embedded in paraffin (6 h) and sliced (8 μm thickness) on a microtome. Staining was done with H&E solution and Masson pine trichrome dye solution. Dyed slices were studied by fluorescence microscopy.

### Swelling tests

Each of the 24 samples (see above) was weighed (W_0_), and subsequently placed in a 5-mL Eppendorf tube containing 1 mL of PBS; the temperature was maintained at 37 °C. The samples were taken out at different time points (6, 12, 24, 36, 48, and 60 h), dried superficially with a filter paper, and weighed (W_t_). Expansion rates were calculated according to the formula: P_t_ = (W_t_ – W_0_)/W_0_ × 100%.

### Compression tests

A second Teflon mold was used to generate cylindrical material samples with the dimensions: diameter 5.0 mm, height 5.0 mm. Formulations were according to Table 2, and irradiation was done as described above. It was decided not to equilibrate the specimens in PBS or any other aqueous medium, as materials **1**–**8** already have high water contents, which are on par with the water content of cartilage (see also Table 2). It is well known that up to 80% of cartilage is water^36^. Then, the samples were compressed using an electronic universal material testing machine (Instron 5944, Norwood, MA, USA), using a crosshair speed of 2 mm/min. Stress-strain curves were measured and Young’s modulus was abstracted as the slope of the curve in the 5–10% strain region^37^. Experiments were done fivefold per composition.

### Degradation tests

Samples had the geometry as described in Swelling tests. Materials were immersed in PBS (24 h, 37 °C); dried superficially, and weighed (W_0_). Then, samples were placed in 0.5 mg/mL collagenase type II and kept at 37 °C. Samples were taken out, dried, weighed (W_t_), and put back at 2, 4, 6, and 8 h. Degradation was quantified using the equation: R_t_ = W_t_/W_0_ × 100%. Experiments were done in triplo.

### Scanning electron microscopy (SEM) analysis—microstructure

Samples of the eight materials were according to the preparation of the hybrid hydrogels (diameter 8.0 mm and height 1.0 mm). First, the specimens were frozen (−80 °C) and then freeze-dried during 48 h (Boyikang Experimental Instrument Co. Ltd., Beijing, China). Samples were cracked and the fracture surfaces were examined using SEM (Hitachi SU 8010, Tokyo, Japan). Prior to the measurements, samples were sputter-coated with Pt, using a Leica EM ACE600 instrument (Leica GmbH, Wetzlar, Germany).

### Cell cultures

Dental Pulp Stem Cells (DPSCs, Stem Cell Bank, Chinese Academy of Sciences, Beijing) were cultured in the α-minimum eagle’s medium (α-MEM) with 10% (v/v) fetal bovine serum (FBS) and 1% (v/v) penicillin/streptomycin. The cells were cultured in a 37 °C incubator with a 5% volume fraction of CO_2_, and the cell culture medium was replaced every 2–3 days. These cells were used in our in vitro experiments.

### Cytotoxicity tests

Formulations **1**–**8** (see Table 2) were added to a 24-well plate (three wells per composition). The plate was then irradiated with UV light (365 nm, 18 mW/cm^2^, 2 min), which leads to photo cross-linking at the bottom of each well. Alcohol (75%) was pipetted into each well to fill it for ~50% and left for 2 h. Then the wells were washed with PBS (3×, 30 min standing for each wash step). DPSCs (5 × 10^3^) were added to each well, and the plate was placed in a 37 °C incubator. Quantitative-iT^Tm^ PicoGreen^®^ dsDNA assay kit was used to detect the proliferation activity of DPSCs seeded onto each of the hybrid hydrogel materials. At 3, 7, and 14th days after the cells were seeded, the DNA content was determined by measuring the UV absorbance (520 nm) with a microplate reader.

### Cellular morphology

Live-dead cell staining and cytoskeleton staining were performed at 1, 3, 7, and 14 days after inoculation. The AO and EB solutions (1:1) in the Live-Dead staining kits were mixed in the dark, following instructions of the supplier. Each well was washed three times with PBS, incubated with AO/EB working solution, observed by fluorescence microscopy, and photographed. Specimens which were used to study the DPSC cytoskeletons were washed with PBS (3×), permeated with 0.1% Triton-X 100, stained with rhodamine and DAPI, observed by fluorescence microscopy, and photographed.

### Cell Differentiation

The main markers of chondrogenic and hypertrophic differentiation include *ACAN*, *Sox9*, *Coll2*, *Col2α1*, *ALP*, and *Col 10A1*. The change of expression level of these specific markers is an important indicator of chondrogenic differentiation. DPSCs were cultured for 7, 14, or 21 days. Total RNA was extracted by Trizol and reverse transcribed into cDNA. These marker genes were subjected to a real-time polymerase chain reaction (RT-PCR) using SYBR Green Master Mix. The primer sequences are listed in Table 3. Real-time PCR was performed at 95 °C for 15 min, and then it was denatured at 95 °C for 10 s, extended at 60 °C for 15 s, and annealed at 72 °C for 15 s, and was cycled 40 times. The melting curve is prepared from 75 °C to 95 °C with a temperature increase of 1 °C every 20 s. The *GAPDH* gene is used as an internal reference gene and normalized expression level, and the ΔΔCt method is used to calculate the relative expression level of the gene. In addition, the ability of the material to induce stem cell chondrogenic differentiation was further verified by Safranin O and Alcian blue staining, as presented in the supplementary material.

**Table 3:** Primer sequences for RT-PCR analysis.

Gene	Forward primer (5′-3′)	Reverse primer (5′-3′)
*Sox9*	AGCGAACGCACATCAAGAC	CTGTAGGCGATCTGTTGGGG
*Col2α1*	CCAGATGACCTTCCTACGCC	TTCAGGGCAGTGTACGTGAAC
*ACAN*	ACTCTGGGTTTTCGTGACTCT	ACACTCAGCGAGTTGTCATGG
*Coll2*	CCAGATGACCTTCCTACGCC	TTCAGGGCAGTGTACGTGAAC
*ALP*	GGAACTCCTGACCCTTGACC	CCACCATCTCGGAGAGTGAC
*Col 10A1*	GCTTCA GGG AGT GCC ATC ATC	CTCACA TTG GAG CCA CTA GGA ATC
*GAPDH*	GGCACAGTCAAGGCTGAGAAT	ATGGTGGTGAAGACGCCAGT

### Qualitative characterization of chondrogenic differentiation

Safranin O and Alcian blue staining was performed at 1, 7, and 14 days after incubation. The samples were washed with PBS (3×) and fixed with 4% neutral formaldehyde, then dyed according to the instructions. After dyeing, washed with PBS (3×) and observed by microscopy and photographed. The results were listed in Supplementary materials.

### Animal experiments

All applicable international, national, and/or institutional guidelines for the care and use of animals were followed. The animal experimental plan of this study was approved by the Ethical Committee of Wenzhou Institute, University of Chinese Academy of Sciences (WIUCAS20033115 and 20200331). Adult male Sprague-Dawley rats (20, weight ~260 g) from Wenzhou Medical University were used to study the utility of the hydrogels (see Table 2) **5** (15%GelMA/1%HAMA), **6** (15%GelMA/1%HAMA/3%DCM), **7** (15%GelMA/ 1%HAMA/6% DCM), and **8** (15%GelMA/1%HAMA/12%DCM) in the rat knee cartilage repair model as described by Cao Tong et al.^38^ with some modifications. **Series B** were first photo-polymerized into circular disks with a diameter of 8.0 mm and a height of 2.0 mm as described above. Then, cylindrical specimens with a diameter of 2.0 mm were cut with a cork drill. Knee cartilage defects with a cylindrical shape (diameter 2.0 mm and depth of 2.0 mm) were created in the right hind leg knee of each animal. Animals were anesthetized with 10% chloral hydrate (4 μL/g). Animals were housed under normal conditions, in the Vivarium facilities of Wenzhou Institute, University of Chinese Academy of Sciences with appropriate feeding and water ad libitum. Specimens **5**, **6**, **7**, or **8** were carefully inserted into the defects (each material in four animals); four animals were used as sham controls. Surgical wounds were carefully closed and penicillin was injected to prevent inflammation^39^. The animals were sacrificed (chloral hydrate, 8 μL/g) after 9 weeks. Specimens and surrounding tissues were excised, photographed, and stored in 10% neutral formalin at 4 °C until histopathological analysis.

### International cartilage repair society (ICRS) analyses

At predetermined time points, samples from each group were graded blindly by two independent observers in terms of cartilage repair according to the international cartilage repair society (ICRS) scoring system (Table 4)^40,41^.

**Table 4:** ICRS macroscopic evaluation of cartilage repair.

Cartilage repair evaluation	Points
Degree of defect repair	
Completely repair (in level with surrounding cartilage)	4
75% cartilage repair	3
50% cartilage repair	2
25% cartilage repair	1
0% cartilage repair	0
Macroscopic appearance	
Intact smooth surface	4
Fibrillated surface	3
Small cracks	2
Large cracks	1
Total degeneration of grafted area	0
Degree of marginal integration	
Complete integration with surrounding cartilage	4
Demarcating border <1 mm	3
3/4 of graft integrated and border >1 mm width	2
1/2 of graft integrated and border >1 mm width	1
From no contact to 1/4 of graft integrated with surrounding cartilage	0
Overall repair assessment	
Grade I: normal	12
Grade II: nearly normal	11–8
Grade III: abnormal	7–4
Grade IV: severely abnormal	3–1

### Histopathology

The samples were fixed overnight in 10% neutral buffered formalin. Then, the samples were decalcified (EDTA 0.1 g/mL, PBS 2 L, Sodium hydroxide 11 mg/mL, pH 7.2, decalcification for 1 month, the supernatant was refreshed every 2 days), sealed with paraffin and sliced using a microtome (Histotome, Leica RM2265, Wetzlar, Germany) to obtain the tissue thickness of 5 µm. The slices were mounted onto microscopic glass and stained with H&E or Safranin O Fast Green following the standard procedures as stated in the instructions. Safranin O Fast Green allowed us to distinguish bone (blue or green) from cartilage (red). The dyed slices were studied in detail with light microscopy and fluorescence microscopy; extensive photography was used to document the results.

### Statistical analysis

Statistics were performed with GraphPad Prism 5 software (GraphPad Software, San Diego, California). Group sizes are specified for each data set, and data were presented as mean ± standard deviation. Statistical significance and *p* levels are indicated as *p < 0.05, **p < 0.01, and ***p < 0.001.

### Reporting summary

Further information on research design is available in the Nature Research Reporting Summary linked to this article.

## Supplementary information

Supplementary Information

Reporting Summary

## Data Availability

The data that support the findings of this study are available from the corresponding author upon reasonable request.
