# Author Correction: The potential utility of hybrid photo-crosslinked hydrogels with non-immunogenic component for cartilage repair

**DOI:** 10.1038/s41536-022-00273-0

**Published:** 2022-12-20

**Authors:** Yili Wang, Levinus Hendrik Koole, Chenyuan Gao, Dejun Yang, Lei Yang, Chunwu Zhang, Huaqiong Li

**Affiliations:** 1 Joint Centre of Translational Medicine, The First Affiliated Hospital of Wenzhou Medical University, Wenzhou, People’s Republic of China; 2 Zhejiang Engineering Research Center for Tissue Repair Materials, Joint Centre of Translational Medicine, Wenzhou Institute, University of Chinese Academy of Sciences, Wenzhou, Zhejiang People’s Republic of China; 3 School of Biomedical Engineering, School of Ophthalmology & Optometry and Eye Hospital, Wenzhou Medical University, Wenzhou, Zhejiang Province People’s Republic of China; 4 Orthopaedic Institute, The First Affiliated Hospital, Soochow University, Suzhou, People’s Republic of China

**Keywords:** Tissue engineering, Implants

Correction to: *npj Regenerative Medicine* 10.1038/s41536-021-00166-8, published online 10 September 2021

In this article, the authors found errors in Figure 1a and Figure 3 that were caused by the accidental mislabelling of the raw images acquired from the scanning electron microscopy experiments. These images were intended to illustrate the structural features of the biomaterials; however, they did not impact the main conclusions of the study. These figures should have appeared as shown below.
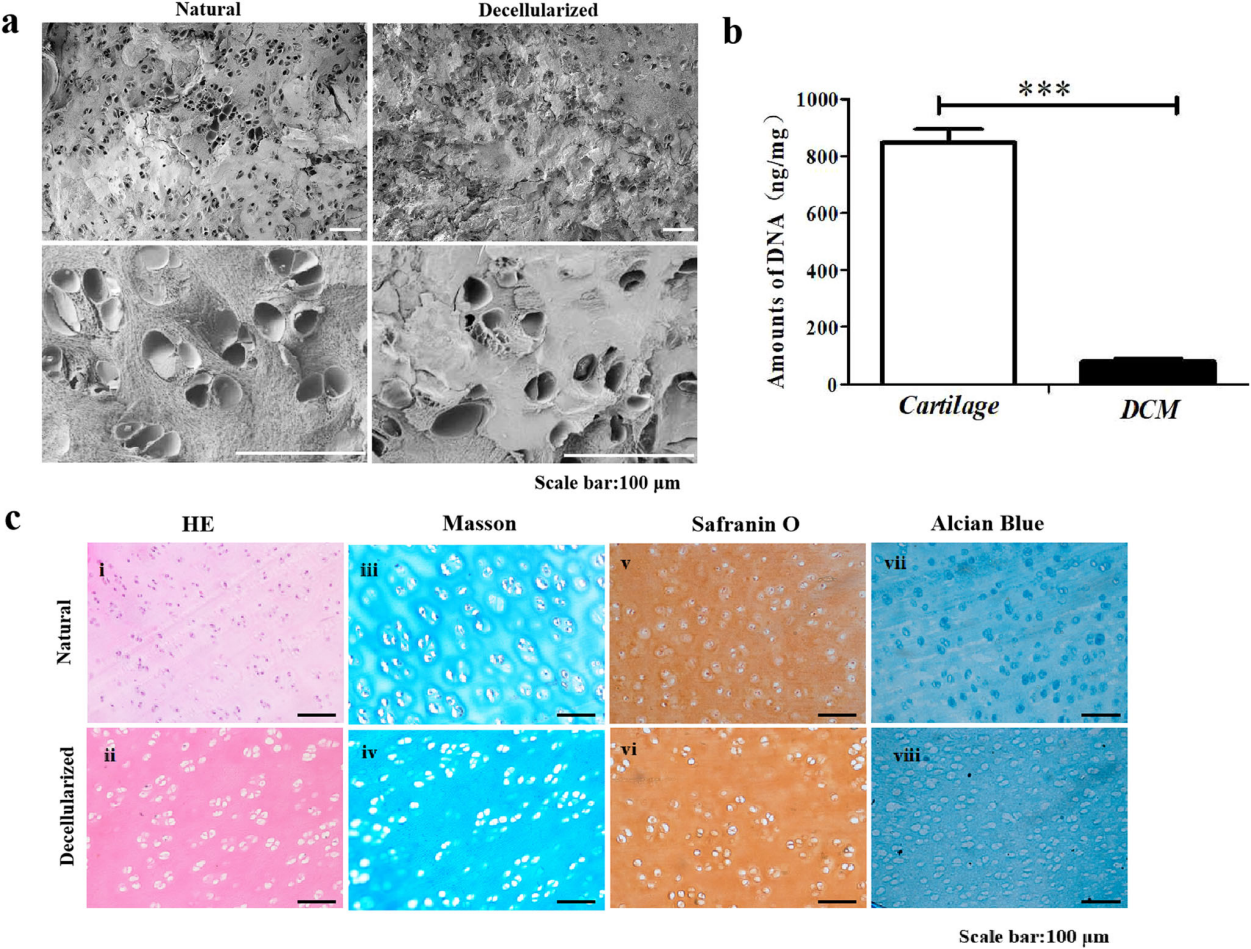

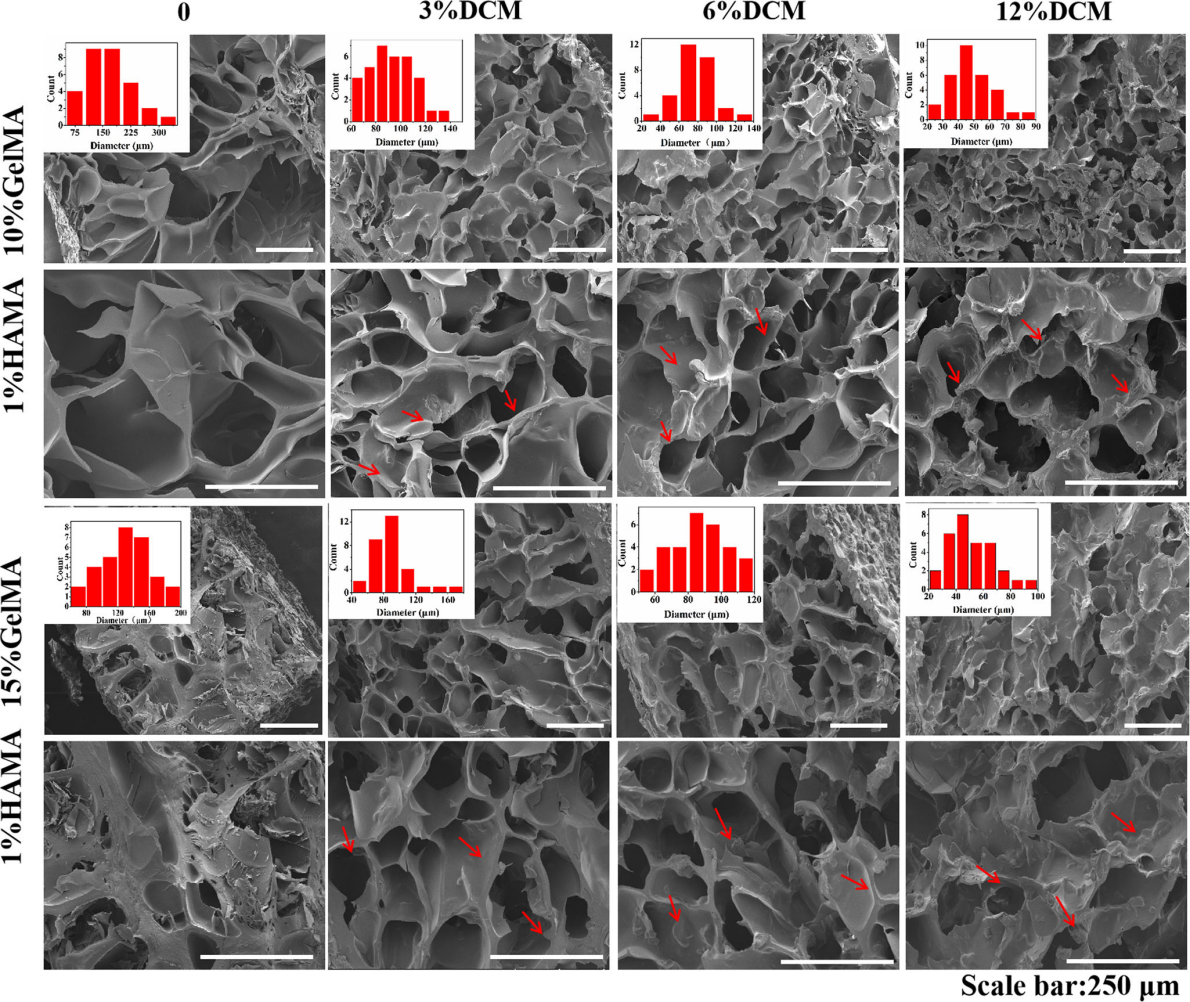


The original article has been corrected.

