# Supplementary material for: The potential utility of hybrid photo-crosslinked hydrogels with non-immunogenic component for cartilage repair
Source: NPJ Regen Med. 2021 Sep 10;6:54. doi: 10.1038/s41536-021-00166-8 (PMC8433347; doi:10.1038/s41536-021-00166-8)
Supplement: Supplementary file 1 — Supplementary Information [file 41536_2021_166_MOESM1_ESM.pdf]

The Potential Utility of Hybrid Photo-crosslinked Hydrogels with Non-immunogenic Component for Cartilage Repair

Yili Wang[1, 2], Levinus Hendrik Koole[3 *], Chenyuan Gao[2], Dejun Yang[2, 3], Lei Yang[4], Chunwu Zhang[1], Huaqiong Li[1, 2 *]

[1] Joint Centre of Translational Medicine, The First Affiliated Hospital of Wenzhou Medical University, Wenzhou, 325035, P.R. China.

[2] Zhejiang Engineering Research Center for Tissue Repair Materials, Joint Centre of Translational Medicine, Wenzhou Institute, University of Chinese Academy of Sciences, Wenzhou, Zhejiang 325000, P.R. China.

[3] School of Biomedical Engineering, School of Ophthalmology & Optometry and Eye Hospital, Wenzhou Medical University, Wenzhou, Zhejiang Province, 325035, P.R. China.

[4] Orthopaedic Institute, The First Affiliated Hospital, Soochow University, Suzhou 215006, P.R. China.

Email: lihq@ucas.ac.cn, leo.koole@eye.ac.cn

Supplementary Results

NMR analysis of GelMA and HAMA

Supplementary Figure 1 shows the 500 MHz ^{1}H NMR spectra of Gel and GelMA (upper panel), and HA and HAMA (lower panel). Note that the spectra of Gel and GelMA are virtually identical, except for the singlet signals which appear at 5.71 and 5.49 ppm [1, 2]. These can be typically attributed to the vinyl protons of the tethered methacrylate groups (the vinyl protons have slightly different chemical shifts). Likewise, the ^{1}H NMR spectra of HA and HAMA are identical except for the singlet peaks; these are found at 5.69 and 6.12 ppm. The appearance of the typical singlet signals, combined with the fact that the materials were extensively dialysed in order to remove low-molecular mass molecules from the reaction mixtures, reveals that attaching methacrylate groups covalently to HA and Gel was successful. Note, furthermore, that NMR could not be used to characterize the hybrid hydrogels **Series A** and **Series B**.

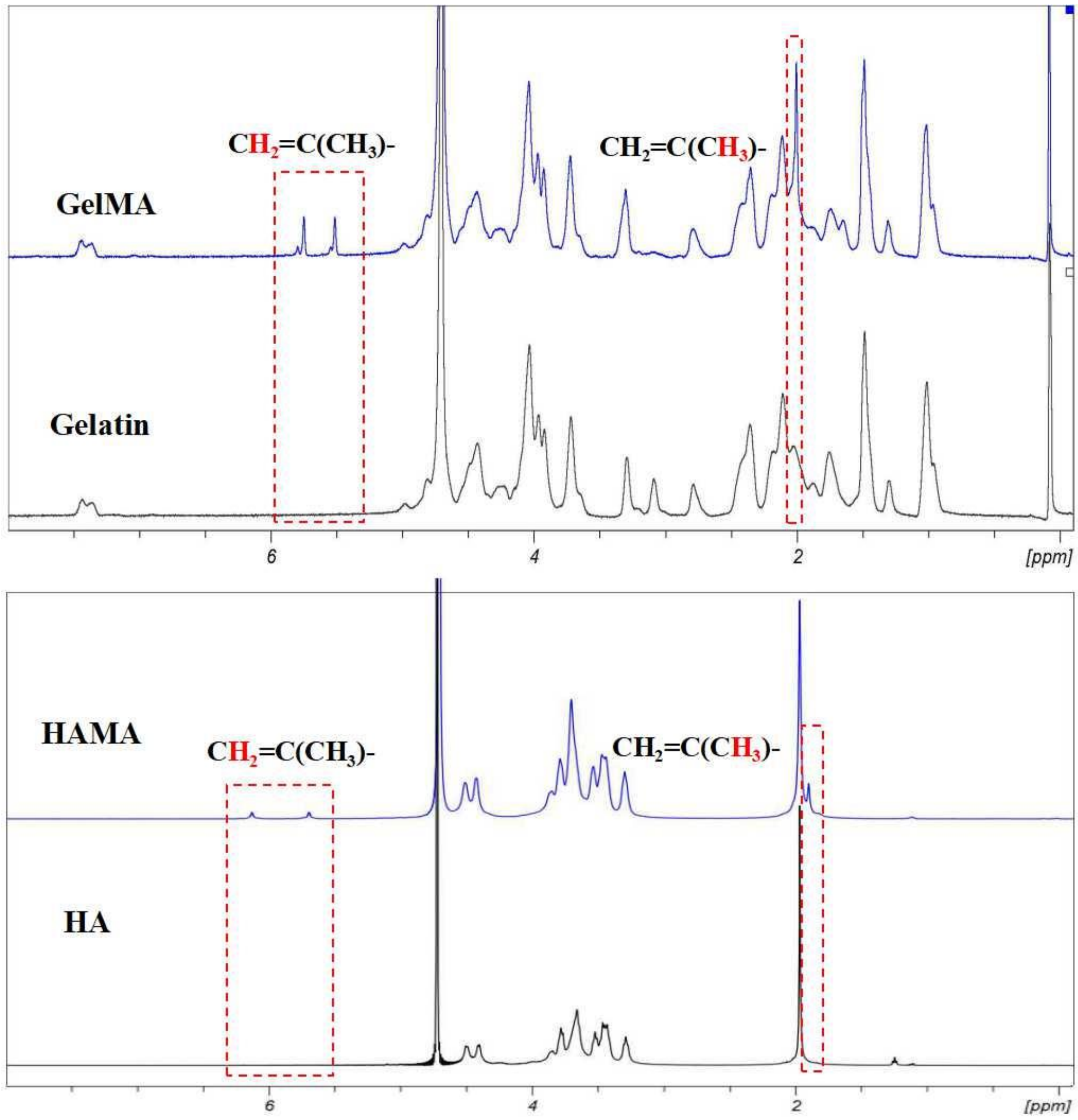

Supplementary Figure 1. ^{1}H NMR (500 MHz) spectra of Gel and GelMA (top panel). Note that the spectra are almost identical, except for the appearance of two singlet signals between 5.5 and 6 ppm in the spectrum of GelMA. These peaks can be attributes to tethered methacrylate groups which are required to engage Gel in photopolymerization/photocuring. The lower panel analogously shows the ^{1}H NMR spectra of HA and HAMA.

FTIR analysis

The FTIR spectra of HAMA, GelMA, the DCM and hydrogel **8** (15%GelMA/1%HAMA /12%DCM) are shown in Supplementary Figure 2. In the spectrum of GelMA, typical amide I and amide II bands are seen in the region 1700-1500 cm^{-1}, revealing its protein nature [3]. The infrared spectrum of DCM is virtually

devoid of typical resonances, except for a broad phosphate-related band occurring at approximately 1000 cm^{-1}. There is a good resemblance between the spectra of the hybrid hydrogel 15%GelMA/1%HAMA/12%DCM on one hand and GelMA on the other hand. The characteristics of HAMA do not clearly show up in the spectrum of the hydrogel, which is in agreement with the fact that the mass content of Gel is approximately 15x higher than the mass content of HA. The spectrum of the hydrogel

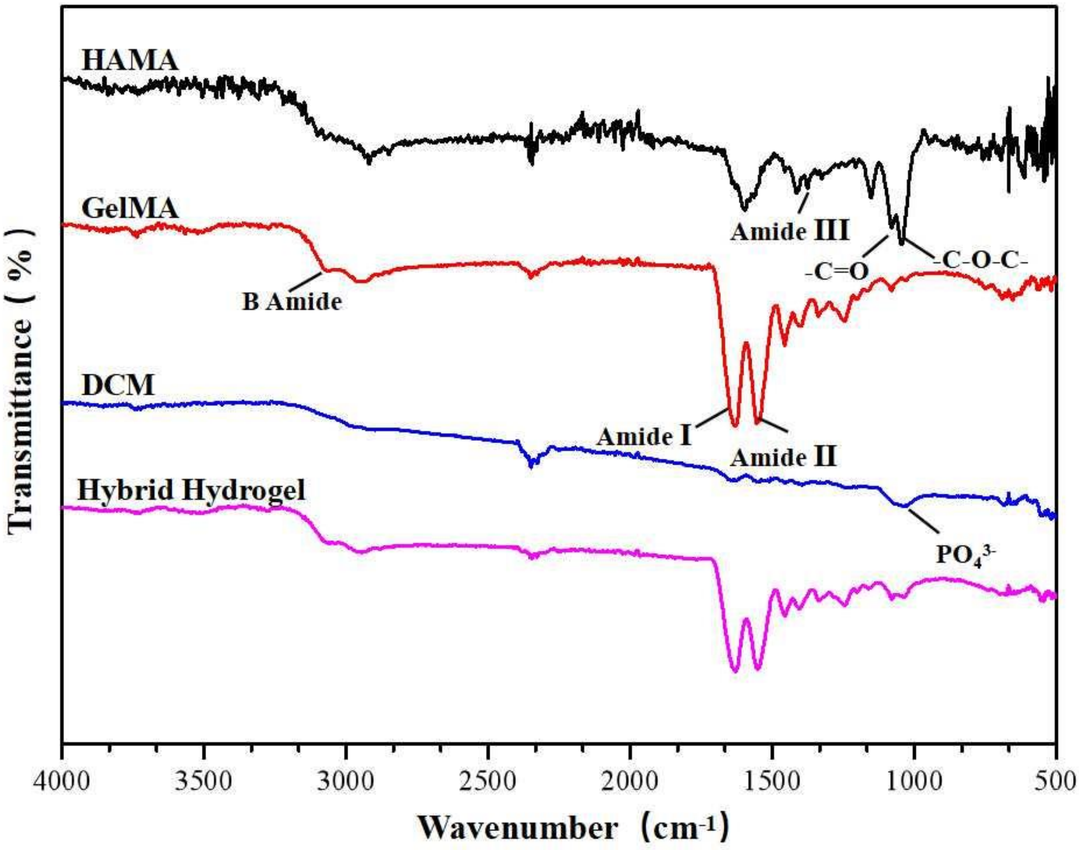

does contain the phosphate band due to DCM (around 1100 - 1000 cm^{-1}).

Supplementary Figure 2. FTIR spectrum spectra of HAMA, GelMA, DCM and hybrid hydrogel. Note the similarity of the spectra of the hydrogel and GelMA. As a marked distinction, the GelMA spectrum features a broad band at 1050 cm^{-1}, which can be attributed to the presence phosphate groups.

Qualitative characterization of chondrogenic differentiation - Safranin O staining & Alcian blue staining

Basophilic cartilage appears red in combination with the Safranin O, and thus can be stained to detect chondrogenic differentiation. As a specific indicator of chondrogenic

differentiation, the GAG combined with Alcian blue will be colored blue. It can be a qualitative characterization of the amount of GAG, and determined the degree of chondrogenic differentiation. Materials **1 - 8** present as thin surface coatings on well-bottoms of a 24-well plate (vide supra), were incubated with DPSCs as described above. Following the Safranin O and Alcian blue protocol, we observed by microscopy. After 1, 7 and 14 days, the color gradually deepened, and the color increased with the increase of DCM. After 14 days, the color of material **8** was the strongest, whereas after 1 day, the material **1** was the weakest (Supplementary Figure 3). The results indicated that the ability to induce chondrogenic differentiation was increased with time and the proportion of DCM, which was elevated the most in material **8**.

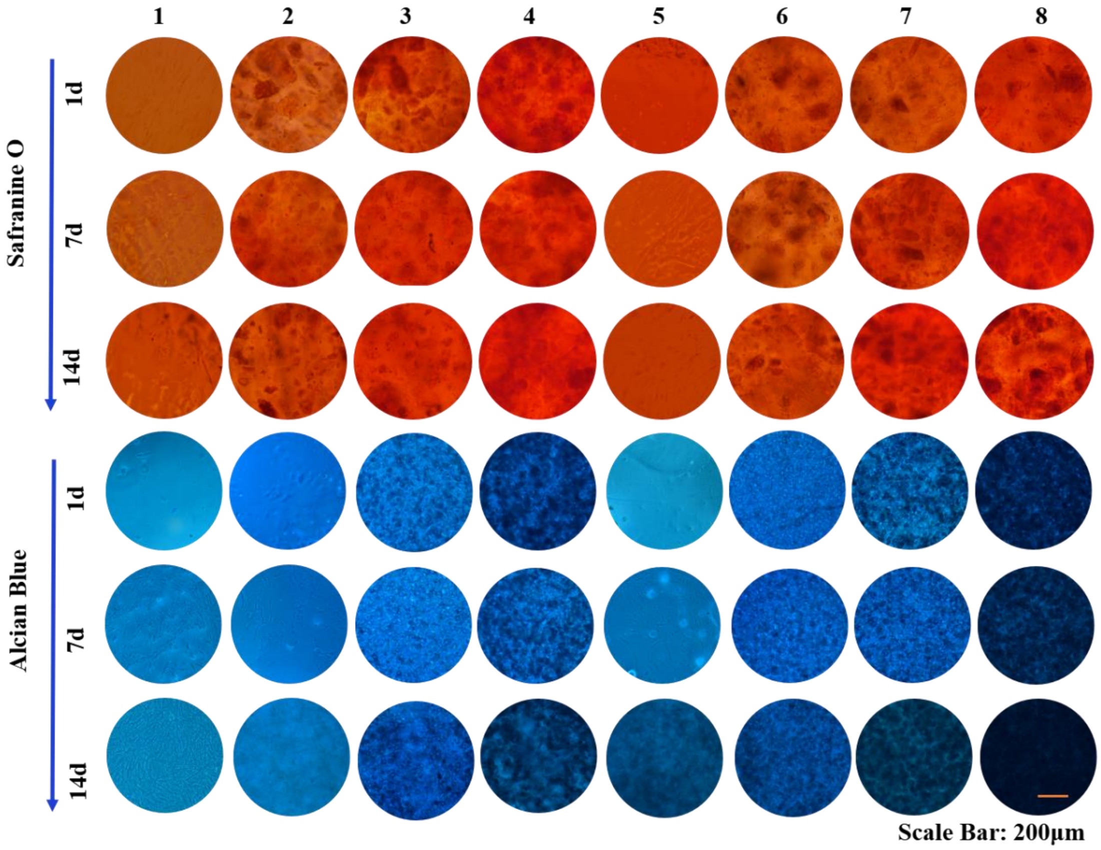

Supplementary Figure 3. Photo micrographs, taken by microscopy, detection of chondrogenic differentiation of cells in contact with the materials of this study; Safranin O and Alcian blue assay. Horizontal: different materials (**1 - 8**); Vertical: time (1, 7 and 14 days post incubation). Scale bar = 200 μm.

Supplementary References

[1] B.Y. Choi, E.P. Chalisserry, M.H. Kim, H.W. Kang, I.W. Choi, S.Y. Nam, The Influence of Astaxanthin on the Proliferation of Adipose-derived Mesenchymal Stem Cells in Gelatin-Methacryloyl (GelMA) Hydrogels, Materials 12 (15) (2019) 2416-2428.

[2] J. Zheng, M. Zhu, G. Ferracci, N.-J. Cho, B.H. Lee, Hydrolytic Stability of Methacrylamide and Methacrylate in Gelatin Methacryloyl and Decoupling of Gelatin Methacrylamide from Gelatin Methacryloyl through Hydrolysis, Macromol. Chem. Phys. 219 (18) (2018) 1800266.

[3] S. Oktay, N. Alemdar, Electrically controlled release of 5-fluorouracil from conductive gelatin methacryloyl-based hydrogels, J. Appl. Polym. Sci. 136 (1) (2019) 46914-46922.
