# Supplementary material for: The potential utility of hybrid photo-crosslinked hydrogels with non-immunogenic component for cartilage repair
Source: NPJ Regen Med. 2021 Sep 10;6:54. doi: 10.1038/s41536-021-00166-8 (PMC8433347; doi:10.1038/s41536-021-00166-8)
Supplement: Supplementary file 2 — Reporting Summary [file 41536_2021_166_MOESM2_ESM.pdf]

nature research

Corresponding author(s): Huaqiong Li, Levinus Hendrik Koole

Last updated by author(s): Aug 18, 2021

Reporting Summary

Nature Research wishes to improve the reproducibility of the work that we publish. This form provides structure for consistency and transparency in reporting. For further information on Nature Research policies, see our Editorial Policies and the Editorial Policy Checklist.

Statistics

For all statistical analyses, confirm that the following items are present in the figure legend, table legend, main text, or Methods section.

n/a	Confirmed	
☐	☒	The exact sample size (*n*) for each experimental group/condition, given as a discrete number and unit of measurement
☐	☒	A statement on whether measurements were taken from distinct samples or whether the same sample was measured repeatedly
☐	☒	The statistical test(s) used AND whether they are one- or two-sided<br>*Only common tests should be described solely by name; describe more complex techniques in the Methods section.*
☒	☐	A description of all covariates tested
☐	☒	A description of any assumptions or corrections, such as tests of normality and adjustment for multiple comparisons
☐	☒	A full description of the statistical parameters including central tendency (e.g. means) or other basic estimates (e.g. regression coefficient) AND variation (e.g. standard deviation) or associated estimates of uncertainty (e.g. confidence intervals)
☒	☐	For null hypothesis testing, the test statistic (e.g. *F*, *t*, *r*) with confidence intervals, effect sizes, degrees of freedom and *P* value noted<br>*Give P values as exact values whenever suitable.*
☒	☐	For Bayesian analysis, information on the choice of priors and Markov chain Monte Carlo settings
☒	☐	For hierarchical and complex designs, identification of the appropriate level for tests and full reporting of outcomes
☒	☐	Estimates of effect sizes (e.g. Cohen's *d*, Pearson's *r*), indicating how they were calculated

Our web collection on statistics for biologists contains articles on many of the points above.

Software and code

Policy information about availability of computer code

Data collection	NMR spectra was run on a Bruker Avance Neo Spectrometer Durham. FTIR spectra were recorded with a Fourier infrared spectrometer instrument (Bruker, Tensor II, Bremen, Germany). Microstructure were examined using SEM (Hitachi SU 8010, Tokyo, Japan). The DNA content was determined with a microplate reader.The gene expression results was obtained useing the fluorescent quantitative PCR instrument (LightCycler® 96,Roche, Switzerland). The cellular morphology was observed by fluorescence microscopy(Nikon,Germany).
Data analysis	The data analysis were performed using Graphpad Prism and OriginPro. All the analysis tools are avaliable upon request.

For manuscripts utilizing custom algorithms or software that are central to the research but not yet described in published literature, software must be made available to editors and reviewers. We strongly encourage code deposition in a community repository (e.g. GitHub). See the Nature Research guidelines for submitting code & software for further information.

Data

Policy information about availability of data

All manuscripts must include a data availability statement. This statement should provide the following information, where applicable:

- Accession codes, unique identifiers, or web links for publicly available datasets
- A list of figures that have associated raw data
- A description of any restrictions on data availability

Analysis tools are avaliable for purposes of reproducing or extending the analysis and other raw data will be avaliable on general request.

Field-specific reporting

Please select the one below that is the best fit for your research. If you are not sure, read the appropriate sections before making your selection.

☒ Life sciences ☐ Behavioural & social sciences ☐ Ecological, evolutionary & environmental sciences

For a reference copy of the document with all sections, see nature.com/documents/nr-reporting-summary-flat.pdf

Life sciences study design

All studies must disclose on these points even when the disclosure is negative.

Sample size	The sample size was determined according to the requirements of the experiment, such as cell experiment, where the sample is prepared in the plate, and the sample size is determined by the size of the plate. Sample sizes used in animal experiments were based on reports in the literature.
Data exclusions	No data were excluded for analysis.
Replication	All the experiments were successfully repeated with similar results.
Randomization	No specific randomization method was used, samples were processed and allocated according to specific experimental conditions and treatments.
Blinding	No blinding was used.

Reporting for specific materials, systems and methods

We require information from authors about some types of materials, experimental systems and methods used in many studies. Here, indicate whether each material, system or method listed is relevant to your study. If you are not sure if a list item applies to your research, read the appropriate section before selecting a response.

Materials & experimental systems

n/a	Involved in the study	
☒	☐	Antibodies
☐	☒	Eukaryotic cell lines
☒	☐	Palaeontology and archaeology
☐	☒	Animals and other organisms
☒	☐	Human research participants
☒	☐	Clinical data
☒	☐	Dual use research of concern

Methods

n/a	Involved in the study	
☒	☐	ChIP-seq
☒	☐	Flow cytometry
☒	☐	MRI-based neuroimaging

Eukaryotic cell lines

Policy information about cell lines

Cell line source(s)	Dental Pulp Stem Cell was purchased from Stem Cell Bank(Chinese Academy of Sciences, Beijing) .
Authentication	Cell lines were tested using Live-Dead and cellular morphology staining, and RT-PCR analysis to confirm changes in nucleic acid level.
Mycoplasma contamination	The cell lines were not tested for mycoplasma contamination.
Commonly misidentified lines (See ICLAC register)	No commonly misidentified cell lines were used.

Animals and other organisms

Policy information about studies involving animals; ARRIVE guidelines recommended for reporting animal research

Laboratory animals	Adult male Sprague-Dawley rats (2 months, weight approximately 260 g) were used as laboratory animals.
Wild animals	The study did not involve wild animals.
Field-collected samples	The study did not involve samples collected from the field.

Ethics oversight	The animal experimental was approved by the Ethical Committee of Wenzhou Institute, University of Chinese Academy of Sciences (WIUCAS20033115, 20200331).

Note that full information on the approval of the study protocol must also be provided in the manuscript.
